# Cardiac pseudoaneurysms: a clinical case series

**DOI:** 10.1093/ehjcr/ytad636

**Published:** 2023-12-23

**Authors:** Grace Alexander, Mahi L Ashwath

**Affiliations:** Department of Internal Medicine, University of Iowa Hospitals and Clinics, Iowa City, IA, USA; Division of Cardiovascular Medicine, Department of Internal Medicine, University of Iowa Hospitals and Clinics, 200 Hawkins Dr, Iowa City, IA 52242, USA

**Keywords:** Cardiac pseudoaneurysm, Cardiac magnetic resonance, Case report, Multi-modality imaging

## Abstract

**Background:**

Cardiac pseudoaneurysms are a potentially life-threatening pathology with a variety of non-specific clinical manifestations. This case series uniquely shares a collection of rare pathologies with differing preceding risk factors and presentations, with an emphasis on the utility of multi-modality imaging in diagnosis and management.

**Case summary:**

We present three cases of cardiac pseudoaneurysms. Case 1 is a 27-year-old woman with delayed presentation of a traumatic left ventricular pseudoaneurysm (LVP). Case 2 is a 73-year-old man with post-myocardial infarction LVP. Case 3 is a 38-year-old man with left ventricular outflow tract pseudoaneurysm after aortic valve replacement.

**Discussion:**

Cardiac pseudoaneurysms are rare and important to diagnose in a timely manner. Advances in non-invasive imaging modalities have improved our ability to distinguish pseudoaneurysms from other pathologies, leading to more timely management.

**Learning points**

- Recognize the variety of aetiologies of cardiac pseudoaneurysms including trauma, post-myocardial infarction, and post-valve replacement.
- Highlight the importance of multi-modality imaging in diagnosis and management of cardiac pseudoaneurysms.

## Introduction

Pseudoaneurysms of the heart are rare but associated with high mortality. Anatomically, a pseudoaneurysm is dangerous because it is an outpouching with a single-layer outer wall that is prone to expanding and rupturing. This contrasts with true aneurysms, which are sturdier outpouchings containing all tissue and muscle layers. Timely diagnosis of pseudoaneurysms is important to guide urgent surgical intervention. In-depth knowledge about the development and progression of pseudoaneurysms is relatively limited due to its low incidence. In one of the larger reported cohorts of 52 patients with cardiac pseudoaneurysms, 48% of cases were discovered incidentally in asymptomatic patients, while the remaining cases had non-specific presentations such as heart failure, chest pain, syncope, and arrythmias.^1^ Given the non-specific clinical signs and high risk of rupture of pseudoaneurysms, imaging plays an essential role in diagnosis. Here, we review three distinct patient cases that demonstrate the importance of keeping cardiac pseudoaneurysms in the differential of non-specific cardiac presentations, as well as the importance of multi-modality imaging in diagnosis and management.

## Summary figure

**Table:** 

	Case 1	Case 2	Case 3
Preceding risk factor	Blunt chest trauma	Myocardial infarction	Surgical aortic valve replacement
Location of lesion	Basal inferolateral, adjacent to mitral valve	Apical septum	Medial aspect of the left ventricular outflow tract
Time to diagnosis	7 years	<1 month	2 months
Diagnostic imaging	CMR	CMR	CCTA
	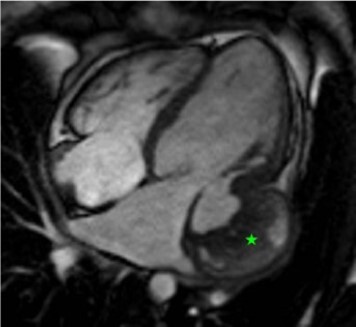	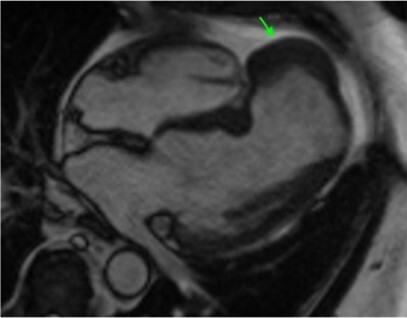	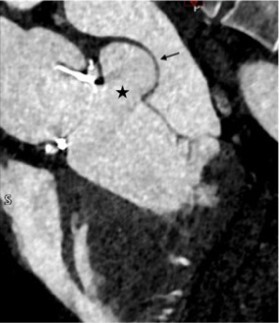

## Case 1

**Figure 1:**
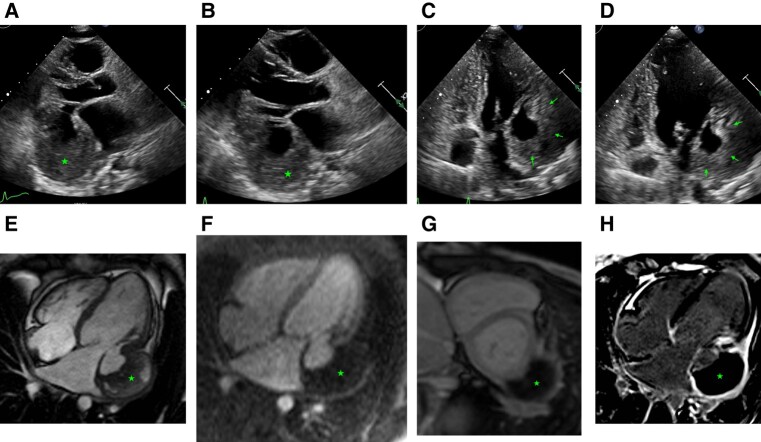
Transthoracic echocardiogram parasternal long-axis view in systole (*A*) and diastole (*B*) revealed a 3.8 × 6.8 cm thickening in the lateral wall of the left ventricle concerning for infiltrative process vs. tumour, indicated by the star. Apical four-chamber view demonstrates this, in systole (*C*) and diastole (*D*), indicated by the arrow. Cardiac magnetic resonance imaging was done on a Siemens Aera 1.5T scanner. Cine sequences were done using steady-state free precession sequences with 0.6 mm slice thickness and 0.4 mm gap. Delayed enhancement sequences were done using high TI imaging with a TI of 600 ms for thrombus evaluation and low TI imaging with a TI of 320 ms, adjusted to the null time, for delayed enhancement and scar imaging. Four-chamber view cine (*E*) shows large left ventricular basal inferolateral pseudoaneurysm, adjacent to the mitral valve, measuring 3.3 cm at the mouth, 4 cm in depth, and 5.9 at the largest dimension, with dense material filling the pseudoaneurysm, suggestive of thrombus (star). Four-chamber perfusion imaging (*F*) showing normal myocardial perfusion and lack of perfusion of the dense material in the pseudoaneurysm, suggestive of a thrombus (star). Short-axis high TI imaging (*G*), with an inversion time of 600 ms, with dark appearance of the dense material in the pseudoaneurysm, consistent with a thrombus (star). Four-chamber delayed enhancement imaging (*H*) showing inferolateral pseudoaneurysm filled with thrombus (star), covered by overlying pericardium.

A 27-year-old female patient with a past medical history of IgA nephropathy and anaemia of chronic disease presented to clinic with shortness of breath, weight gain, leg swelling, inability to lie flat, and palpitations. Vitals were stable and the exam revealed a 3/6 systolic ejection murmur and 2+ bilateral pitting oedema up to the thighs. Transthoracic echocardiogram (TTE) revealed left ventricular ejection fraction (LVEF) of 53% with severe mitral regurgitation, moderate tricuspid regurgitation (see Supplementary material online, *Movies S1–S3*), and a 3.8 × 6.8 cm thickening in the lateral wall of the left ventricle concerning for infiltrative process vs. tumour (*Figure 1A–D*). Based on European Society of Cardiology (ESC) Class I recommendations to obtain cardiac magnetic resonance (CMR) imaging when an infiltrative process is on the differential, CMR was done, which revealed a large basal inferolateral pseudoaneurysm, adjacent to the mitral valve, measuring 3.3 cm at the mouth, 4 cm in depth, and 5.9 cm at the largest dimension, with dense material filling the pseudoaneurysm, suggestive of thrombus (*Figure 1E–H*; Supplementary material online, *Movie S4*). Further review of past medical history revealed a car accident 7 years prior with blunt trauma from the left side. The patient underwent mitral valve repair with bovine pericardial patch augmentation of posterior leaflet and annuloplasty, as well as repair of the chronic atrioventricular groove left ventricular aneurysm with bovine pericardial patch. Five years later, she presented with acute heart failure symptoms and was found to have mitral valve repair graft failure needing mitral valve replacement with a mechanical valve along with a tricuspid valve repair.

## Case 2

**Figure 2:**
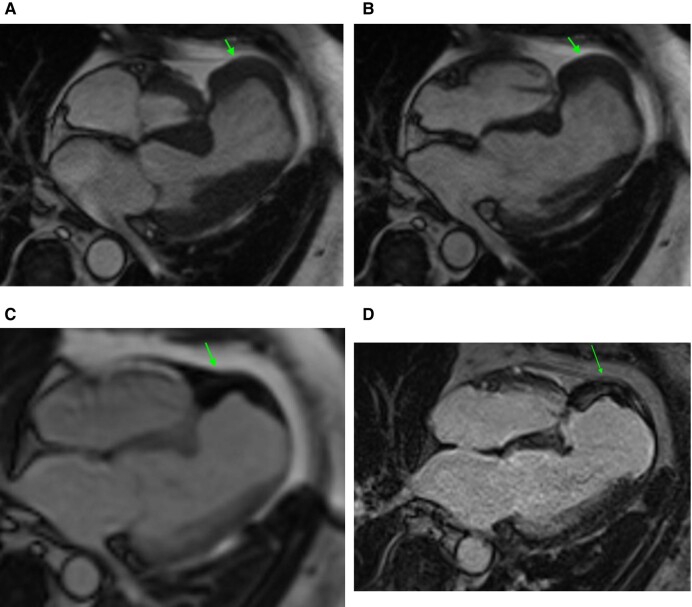
Cardiac magnetic resonance imaging. Pseudoaneurysm in each image is denoted by an arrow. Four-chamber view cine in systole (*A*) and diastole (*B*), with reduced function, measured LVEF of 33% and apical septal pseudoaneurysm. High TI imaging (*C*) with dark appearance of the dense material in the pseudoaneurysm, consistent with a thrombus. Delayed enhancement imaging (*D*) shows pseudoaneurysm containing thrombus at the apex of the left ventricle. The pseudoaneurysm measures 4.4 × 2.8 cm.

A 73-year-old male with a past medical history of hypertension, type 2 diabetes, chronic kidney disease, asthma, and chronic musculoskeletal pains underwent a pre-operative cardiac workup before elective knee surgery. Electrocardiogram showed Q waves in the anterior leads, suggesting prior anteroseptal myocardial infarction (MI) of undetermined age. Pharmacologic stress test revealed a large partially reversible severe perfusion deficit centred at the apex and distal septum extending to the apical segments. A TTE showed a large multi-lobe outpouching at the left ventricular apex, concerning pseudoaneurysm vs. aneurysm (see Supplementary material online, *Movie S5*). The patient denied current or prior history of chest pain, jaw pain, epigastric pain, nausea, or vomiting; however, he noted significant physical activity limitations due to knee pain. He did recall a 3-day period of fatigue, several weeks prior which resolved spontaneously. Physical exam was unremarkable except for 1–2 + pitting oedema. Coronary angiogram showed mid left anterior descending with 99% stenosis, distal left anterior descending artery with 100% stenosis, first diagonal branch with 80% stenosis, and mid left circumflex with 80% stenosis. The leading diagnosis was a left ventricular pseudoaneurysm related to a prior anterolateral MI. CMR was done to confirm TTE findings and assist in surgical planning. CMR showed a pseudoaneurysm with thrombus at the apical septum measuring 4.4 × 2.8 cm, thrombus measuring 4.4 × 1.2 cm, along with reduced left ventricular function, calculated LVEF of 33% (*Figure 2A–D*). The patient underwent pseudoaneurysm repair with patch and coronary artery bypass grafting of a single diagonal vessel. The post-operative course was unremarkable except for brief atrial fibrillation.

## Case 3

**Figure 3:**
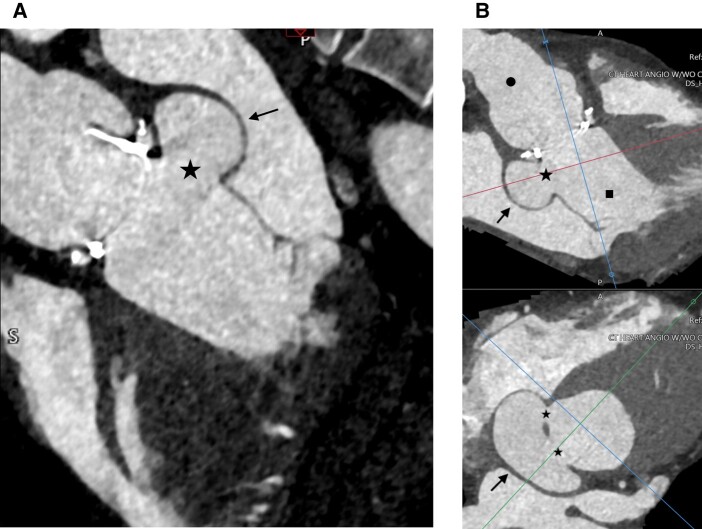
Cardiac computed tomographic angiography imaging. Pseudoaneurysm (arrow) can be seen on the medial aspect of the left ventricular outflow tract body measuring 4.4 cm × 2.2 cm (*A*). The stars indicate the openings to the pseudoaneurysm. The patient’s bioprosthetic aortic valve can be seen just superior to the pseudoaneurysm. Additional views include CCTA showing two openings into the pseudoaneurysm and the corresponding orthogonal plane through each opening into the pseudoaneurysm (*B*), with ascending aorta (circle) above this, and left ventricular outflow tract (square) below.

A 38-year-old African American male presented to clinic for surgical evaluation of an ascending aortic aneurysm. Past medical history was notable for severe aortic insufficiency of the bicuspid aortic valve, status post-aortic valve repair via full sternotomy 19 years earlier. Other medical history included sickle cell trait and Meniere’s disease. Social history included cannabis use. He had yearly TTE (see Supplementary material online, *Movie S6*) and cardiac computed tomography angiography (CCTA) to monitor the aneurysmal dilation of the aortic root, continuing until there was an increase at the sinus of Valsalva from 5.3 to 5.5 cm in the time span of 1 year. Based on his aortic root diameter that was greater than 4.5 cm, coupled with the need for concomitant aortic valve surgery, he underwent repair and valve replacement per ESC guidelines. Specifically, he underwent ascending aortic aneurysm repair with a Bentall graft and aortic valve replacement with a bioprosthetic valve. He was extubated on post-operation Day 2 and discharged home on Day 5. He returned to clinic for follow-up 2 months later. He reported feeling generally well and denied any cardiac symptoms. The CT chest revealed contrast-filled outpouching in communication with the medial aspect of the left ventricular outflow tract (LVOT), measuring up to 4.4 cm × 2.9 cm, raising concern for pseudoaneurysm. TTE found the prosthetic aortic mean gradient to be 7 mmHg and mildly reduced LVEF of 48%. CCTA imaging confirmed LVOT outpouching on the medial aspect of LVOT, with pseudoaneurysm body measuring 4.4 cm × 2.2 cm (*Figure 3A*). The patient was instructed to follow-up in another three months with CCTA to determine if the pseudoaneurysm is changing.

## Discussion

A majority of pseudoaneurysms are sequelae of MIs or cardiothoracic surgery, while a much lesser percentage stem from trauma or infection.^2^ Blunt chest trauma causes rapid deceleration of the body and leads to shearing forces on the heart. Case 1 demonstrates blunt chest trauma as a rare cause of pseudoaneurysm. Also interesting is her delayed presentation after chronic progressive haemodynamic changes. Late presentations of pseudoaneurysms can happen rarely and should be considered in the differential diagnosis. In this case, CMR was done to further evaluate a suspected mass on TTE and instead showed a pseudoaneurysm. The case highlights the limitation of TTE and the need for advanced imaging for suspected cardiac masses or other structural abnormalities.

Case 2 highlights the most common cause of pseudoaneurysm, which is post-MI, when wall necrosis leads to a contained rupture. The diagnosis of pseudoaneurysm can be difficult because the most common presenting symptoms are non-specific including heart failure, chest pain, and dyspnoea^3^; furthermore, there are a substantial number of asymptomatic cases.^1^ The patient in Case 2 had what was suspected to be a silent MI in the setting of his diabetes, only to have pseudoaneurysm incidentally found weeks later. Reports of asymptomatic cases range from 10 to 48% of study cohorts.^1,2^ This is important for clinicians to keep in mind when managing patients with a history of known pseudoaneurysm associations (i.e. MI, surgery, chest trauma, and endocarditis). CMR in this case was useful for further characterization of the pseudoaneurysm, detecting thrombus and guiding surgical intervention—all advantages that CMR has when compared with TTE (*Table 1*).

**Table 1:** The advantages and disadvantages of various imaging modalities: transthoracic echocardiogram, trans-oesophageal echocardiogram, cardiac magnetic resonance imaging, and cardiac computed tomographic angiography

	Advantages	Disadvantages
Transthoracic echo (TTE)	Widely available Easy to obtain bedside Cost-effective Doppler to assess haemodynamics Evaluates both structure and function	Operator dependent Small windows Less tissue characterization Can miss off-axis findings
Trans-oesophageal echo (TEE)	Available at most centres Excellent anatomical and functional images	More invasive and time intensive Needs sedation Needs a cooperative patient
Cardiac magnetic resonance (CMR) imaging	Structural visualization and tissue characterization superior to other modalities Preferred modality for imaging scar, thrombus, and masses Delayed enhancement imaging can provide information not obtainable by other imaging methods	Time-consuming Needs centre and operators with expertise in CMR Expensive Needs a cooperative patient
Cardiac computed tomographic angiography (CCTA)	Quick image acquisition time Large windows 3D capabilities Excellent spatial resolution and anatomical information^4^	No haemodynamic data Uses radiation Uses CT contrast

As seen with Case 3, LVOT pseudoaneurysms can develop after cardiothoracic surgery, such as surgical aortic valve replacement. Additional causes include trauma (deceleration, torsional, and penetrating) and infection such as infectious aortitis.^4^ Transthoracic echocardiogram did not show the pseudoaneurysm, given the need for off-axis imaging (*Figure 3B*). Pseudoaneurysm was detected on CCTA done for routine post-operative follow-up after the Bentall procedure. CCTA plays a central role in the diagnosis, risk stratification, and management of aortic valve diseases—notably with prosthetic valves. CMR can be limited by the metal artefact. CCTA is the preferred method for evaluation for prosthetic aortic valves. Full-beat acquisition of CCTA images helped provide functional information about the pseudoaneurysm (see Supplementary material online, *Movies S7* and *S8*).

TTE is an excellent first-line imaging modality due to wide accessibility, bedside capabilities, cost-effectiveness, Doppler for evaluating haemodynamics, and high sensitivity and specificity for detecting true aneurysms, estimated at 90%.^5^ Unfortunately, TTE struggles with clearly differentiating true aneurysm from pseudoaneurysm. Proposed diagnostic clues for pseudoaneurysms on TTE include a smaller neck-to-body diameter ratio of 0.25–0.5 vs. 0.9–1.0 for aneurysms,^6^ as well as turbulent flow by colour Doppler at the neck/orifice^7^; see *Table 1*. CMR has unparalleled capabilities of detailing the pericardium, myocardium, and epicardial fat layers. An abrupt transition between the healthy and scarred myocardium suggests pseudoaneurysm on CMR.^8^ Delayed enhancement of the outpouching’s overlying pericardium has been shown to be highly sensitive for pseudoaneurysm post-MI^9^; see *Table 2*. When focused on aortic outpouchings, CT is useful for its quick image acquisition time, large windows providing anatomical information, three-dimmensional imaging capabilities, and spatial resolution^4^; see *Table 1*. CCTA is especially indicated for prosthetic valve complications. In general, surgical management with patch closure or primary closure is recommended, especially in patients with symptoms.

**Table 2:** Pseudoaneurysm and true aneurysm features on transthoracic echocardiogram and cardiac magnetic resonance imaging

	Pseudoaneurysm	True Aneurysm
Transthoracic echo (TTE)	Narrow neck: neck to maximal diameter ratio 0.25–0.5^5^ Turbulent flow by pulsed Doppler at the neck of the outpouching^6^	Wide neck: neck to maximal diameter ratio 0.9–1.0^5^
Cardiac magnetic resonance (CMR) imaging	Cine imaging: Narrow neck: neck to maximal diameter ratio 0.25–0.5 Abrupt transition between healthy and scarred myocardium^7^ Delayed enhancement imaging: Enhancement of the overlying pericardium post-MI^8^	Cine imaging: Wide neck: neck to maximal diameter ratio 0.9–1.0 Delayed enhancement imaging: Smooth transition between healthy and scarred myocardium Non-enhancing pericardium post-MI

In conclusion, we have reviewed three cases of cardiac pseudoaneurysms that highlight a variety of aetiologies including trauma, MI, and surgery along with different presentation timelines. Pseudoaneurysms are important to distinguish from true aneurysms, as the former are prone to rupture and often necessitate surgery. In each case, multi-modality imaging assisted with diagnosis and surgical planning.

## Supplementary Material

ytad636_Supplementary_Data

## Data Availability

All data are incorporated into the article and its online Supplementary material.

## Lead author biography

Grace Alexander, MD, is an Internal Medicine resident physician at the University of Iowa Hospitals and Clinics in Iowa City, IA, USA. She completed her medical degree at Rush Medical College in 2022 and undergraduate studies at Indiana University in 2017. Her clinical interests include cardiac imaging and cardio-oncology.

## Supplementary material

**Consent:** The authors confirm that consent for submission of this case report including images and associated text has been obtained from all patients in compliance with COPE guidelines.

**Funding:** None declared.
